# Supplementary material for: A Highly Conserved Region in BRCA2 Suppresses the RAD51-Interaction Activity of BRC Repeats
Source: Vet Sci. 2023 Feb 10;10(2):145. doi: 10.3390/vetsci10020145 (PMC9959916; doi:10.3390/vetsci10020145)
Supplement: Supplementary file 1 [file vetsci-10-00145-s001.zip › Supplemental Table S1.pdf]

Supplemental Table S1. Base sequences of primers used in this study.

		For PCR		For sequencing
1	Forward	cB2 intron 10 1036–1058 nt	5′-CATGTAATTCTTGCTTCAGATAC-3′	cB2 Exon 11 642–618 nt
	Reverse	cB2 exon 11 1152–1129 nt	5′-CTTTATTAGAAGCTGTTTTGAAGC-3′	5′-ACCTTTACTGAAGATGATGCTACTG-3′
2	Forward	cB2 exon 11 883–903 nt	5′-GTACAGACCTGGATGACAAAC-3′	
	Reverse	cB2 exon 11 2121–2094 nt	5′-CAGATTCTGAGTTACAAGTATTTCTACC-3′	
3	Forward	cB2 exon 11 1799–1820 nt	5′-GAATGTTGGTAGTGAAGCATTG-3′	
	Reverse	cB2 exon 11 3053–3031 nt	5′-ATGTACATCAACTTTCTGAGAGG-3′	
4	Forward	cB2 exon 11 2860–2881 nt	5′-GTGAATTAGCTTGTGGGACAAC-3′	
	Reverse	cB2 exon 11 4060–4036 nt	5′-GTTTTTGAATTAATCTGACAAGGTG-3′	
5	Forward	cB2 exon 11 3777–3797 nt	5′-CAGACAAAAGTGACAGAGGG-3′	
	Reverse	cB2 intron 11 143–119 nt	5′-GAAATCACATTCCCCTAAACTATAC-3′	
