# Supplementary material for: A Highly Conserved Region in BRCA2 Suppresses the RAD51-Interaction Activity of BRC Repeats
Source: Vet Sci. 2023 Feb 10;10(2):145. doi: 10.3390/vetsci10020145 (PMC9959916; doi:10.3390/vetsci10020145)
Supplement: Supplementary file 1 [file vetsci-10-00145-s001.zip › Supplemental Table S2.pdf]

Supplemental Table S2. Characteristics and *BRCA2* allele types of mammary tumor samples.

Breeds	Age	Benign/Malignant	Pathological diagnosis	Sex	Contraception	2135G＞A (E643K)	2213A>G (N669D)	2329T>C	2609A>C (K801Q)	2696A>G (I830V)	3538T>G (I1110M)	4481A>C (T1425P)	4512A>G (K1435R)	5788G>A	6886A>G (I2226M)
Chihuahua	10 y 9 m	Benign	Duct papilloma	♀		-	-	-	A/C	-	-	-	G/G	-	-
Miniature Dachshund	6 y	Benign	Adenoma	♀		-	-	-	-	-	-	-	-	-	-
Miniature Dachshund	8 y	Benign	Benign mixed tumor	♀		-	-	-	A/C	-	-	A/C	-	-	-
Miniature Dachshund	8 y	Benign	Benign mixed tumor	♀		-	-	-	A/C	-	-	-	A/G	-	-
Miniature Dachshund	9 y	Benign	Adenoma	♀		-	-	-	C/C	-	-	-	-	-	-
Miniature Dachshund	10 y	Benign	Benign mixed tumor	♀		-	-	-	A/C	-	-	-	A/G	-	-
Miniature Dachshund	9 y 2 m	Benign	Complex adenoma	♀		-	-	-	-	-	-	-	-	-	-
Pomeranian	5 y	Benign	Benign mixed tumor	♀		-	-	-	A/C	-	-	-	A/G	-	-
Pug	6 y 11 m	Benign	Complex adenoma	♀		-	-	-	-	-	-	-	-	-	-
Toy poodle	13 y	Benign	Adenoma	♀	✓	-	-	-	-	-	-	-	A/G	-	-
Unknown	Unknown	Benign	Benign mixed tumor	♀		-	-	-	-	-	-	C/C	-	-	-
Unknown	Unknown	Benign	Benign mixed tumor	♀		-	-	-	C/C	-	-	-	-	-	-
American Cocker Spaniel	7 y 2 m	Malignant	Complex carcinoma	♀		-	-	-	C/C	-	-	-	-	-	-
Corgi	10 y 4 m	Malignant	Squamous cell carcinoma	♀		-	-	-	C/C	-	-	-	-	-	-
Doberman	1 y 10 m	Malignant	Complex and simple adenoma	♀		-	-	-	C/C	-	-	-	-	-	-
Golden retriever	8 y 3 m	Malignant	Complex carcinoma	♀	Unknown	-	-	-	A/C	-	-	-	A/G	-	-
Labrador Retriever	11 y 1 m	Malignant	Benign mixed tumor	♀		-	-	-	A/C	-	-	-	G/G	-	-
Labrador Retriever	12 y 9 m	Malignant	Simple carcinoma	♀		-	-	-	A/C	-	-				

Miniature Dachshund	10 y	Malignant	Complex carcinoma	♀		-	-	-	C/C	-	-	-	-	-	-
Miniature Dachshund	8 y	Malignant	Complex carcinoma	♀		-	-	-	-	-	-	-	G/G	-	-
Miniature Dachshund	9 y 4 m	Malignant	Simple carcinoma	♀		-	-	-	A/C	-	-	-	-	-	-
Miniature Dachshund	9 y 1 m	Malignant	Simple carcinoma	♀		-	-	-	-	-	-	-	-	-	-
Papillon	12 y	Malignant	Complex carcinoma	♀		-	-	-	A/C	-	-	A/C	-	-	-
Pomeranian	13 y 1 m	Malignant	Complex carcinoma	♀		-	-	-	-	-	-	-	-	-	-
Shiba	11 y	Malignant	Simple carcinoma	♀		-	-	C/T	A/C	-	-	-	-	G/A	-
Shih Tzu	8 y 10 m	Malignant	Complex carcinoma	♀		-	-	-	-	-	-	C/C	-	-	-
Shih Tzu	8 y 8 m	Malignant	Simple carcinoma	♀		-	-	-	-	-	-	-	A/G	-	-
Toy poodle	10 y	Malignant	Complex carcinoma	♀		-	A/G	-	-	A/G	-	-	G/G	-	A/G
Mix	13 y	Malignant	Complex carcinoma	♀	✓	-	-	-	-	-	-	-	A/G	-	-
Mix	10 y 2 m	Malignant	Simple carcinoma	♀		-	-	-	-	-	-	-	-	-	-
Mix	10 y 10 m	Malignant	Complex carcinoma	♀		-	-	-	A/C	-	-	-	A/G	-	-
Ainu	10 y	Unknown	–	♀		-	-	-	-	-	-	-	-	-	-
Cavalier King Charles Spaniel	11 y 5 m	Unknown	–	♀		-	-	-	-	-	-	-	G/G	-	-
Cavalier King Charles Spaniel	14 y	Unknown	–	♀		-	-	-	A/C	-	-	-	G/G	-	-
Chihuahua	13 y	Unknown	–	♀		-	-	-	-	-	-	-	-	-	-
English Setter	16 y	Unknown	–	♀	Unknown	-	-	-	-	-	-	-	-	-	-
Golden retriever	6 y	Unknown	–	♀	Unknown	-	-	-	-	-	-	-	G/G	-	-
Golden retriever	7 y 10 m	Unknown	–	♀		-	-	-	-	-	-	-	G/G	-	-
Miniature Dachshund	8 y 5 m	Unknown	–	♀		-	-	-	A/C	-	-	-	-	-	-

Miniature Dachshund	9 y	Unknown	–	♀	Unknown	-	-	-	A/C	-	-	-	-	-	-
Miniature Dachshund	11 y	Unknown	–	♀		-	-	-	A/C	-	-	-	-	-	-
Papillon	6 y	Unknown	–	♀		-	-	-	A/C	-	-	-	-	-	-
Papillon	13 y	Unknown	–	♀		-	-	-	C/C	-	-	-	-	-	-
Pomeranian	Unknown	Unknown	–	♀	Unknown	-	-	-	-	-	-	C/C	A/G	-	-
Poodle	12 y	Unknown	–	♀		-	A/G	-	-	A/G	-	-	G/G	-	A/G
Poodle	14 y	Unknown	–	♀		-	-	-	C/C	-	-	-	-	-	A/G
Pug	8 y	Unknown	–	♀	Unknown	-	-	-	C/C	-	-	-	-	-	-
Pug	8 y	Unknown	–	♀	Unknown	-	-	-	C/C	-	-	-	-	-	-
Shetland Sheepdog	7 y 6 m	Unknown	–	♀	✓	-	-	-	A/C	-	-	-	-	-	-
Shetland Sheepdog	8 y 6 m	Unknown	–	♀		-	-	-	A/C	-	G/G	-	-	-	-
Shetland Sheepdog	11 y	Unknown	–	♀		-	-	-	C/C	-	-	-	-	-	-
Shetland Sheepdog	13 y	Unknown	–	♀		-	-	-	-	-	-	-	-	-	-
Shiba	13 y 7 m	Unknown	–	♀	Unknown	-	-	-	A/C	-	-	-	A/G	-	-
Shih Tzu	12 y	Unknown	–	♀		G/A	-	C/T	-	-	-	-	G/G	-	-
Shih Tzu	16 y	Unknown	–	♀	Unknown	-	-	-	A/C	-	-	-	G/G	-	-
Shih Tzu	7 y 8 m	Unknown	–	♀		-	-	-	-	-	-	-	-	-	-
Toy poodle	14 y	Unknown	–	♀	✓	-	A/G	-	-	A/G	-	-	G/G	-	A/G
Toy poodle	13 y	Unknown	–	♀		-	G/G	-	-	G/G	-	-	-	-	G/G
Mix	11 y	Unknown	–	♀	✓	-	-	-	-	-	-	-	G/G	-	-
Mix	15 y	Unknown	–	♀		-	-	-	C/C	-	-	-	-	-	-

Mix	12 y 8 m	Unknown	–	♀		-	-	C/C	-	-	-	-	G/G	-	-
Mix	11 y	Unknown	–	♀	Unknown	-	-	-	-	-	-	-	G/G	-	-
Mix	Unknown	Unknown	–	♀	Unknown	-	-	-	-	-	-	C/C	-	-	-
Mix	Unknown	Unknown	–	♀	Unknown	-	-	-	-	-	-	C/C	-	-	-
Mix	11 y	Unknown	–	♀	Unknown	-	-	-	-	-	-	-	A/G	-	-
Mix	10 y	Unknown	–	♀	Unknown	-	-	-	-	-	-	-	A/G	-	-
Mix	13 y	Unknown	–	♀	Unknown	-	-	-	A/C	-	-	-	-	G/A	-
Mix	Unknown	Unknown	–	♀	Unknown	G/A	-	C/T	-	-	-	-	G/G	G/A	-
Mix	13 y	Unknown	–	♀		-	-	-	-	-	-	-	G/G	-	-
Unknown	Unknown	Unknown	–	♀	Unknown	-	-	-	A/C	-	-	-	G/G	-	-
Unknown	Unknown	Unknown	–	♀	Unknown	A/A	-	C/C	-	-	-	-	-	A/A	-
Unknown	Unknown	Unknown	–	♀		-	-	-	C/C	-	-	-	-	-	-
