# Supplementary material for: A Highly Conserved Region in BRCA2 Suppresses the RAD51-Interaction Activity of BRC Repeats
Source: Vet Sci. 2023 Feb 10;10(2):145. doi: 10.3390/vetsci10020145 (PMC9959916; doi:10.3390/vetsci10020145)
Supplement: Supplementary file 1 [file vetsci-10-00145-s001.zip › Supplemental Table S3.pdf]

Supplemental Table S3. The characteristics and *BRCA2* allele type in tumor-free dog samples.

Breed	Age	Sex	Castration/ Contraception	2135G＞A (E643K)	2213A>G (N669D)	2329T>C	2609A>C (K801Q)	2696A>G (I830V)	3538T>G (I1110M)	4481A>C (T1425P)	4512A>G (K1435R)	5788G>A	6886A>G (I2226M)
Beagle	2 y	♂		-	-	-	A/C	-	-	A/C	-	-	-
Beagle	Unknown	♂		-	-	-	-	-	-	-	G/G	-	-
Beagle	1 y	♀		-	-	-	C/C	-	-	-	-	-	-
Beagle	1 y	♀		-	-	-	-	-	-	A/C	-	-	-
Beagle	1 y	♀					-	-	-	A/C	-	-	-
Beagle	1 y	♀		-	-	-	-	-	-	-	-	-	-
Beagle	1 y	♀		-	-	-	-	-	-	A/C	-	-	-
Beagle	7 y	♂	✓	-	-	-	C/C	-	-	-	-	-	-
Beagle	4 y	♀		-	-	-	C/C	-	-	-	A/G	-	-
Beagle	9 y	♀		-	-	-	-	-	-	A/C	-	-	-
Beagle	9 y	♀		-	-	-	-	-	-	C/C	-	-	-
Bichon Frise	10 y	♂		-	-	-	-	-	-	-	A/G	-	-
Cavalier King Charles Spaniel	4 y	♀		-	-	-	C/C	-	-	-	-	-	-
Cavalier King Charles Spaniel	6 y	♂		-	-	-	C/C	-	-	-	-	-	-
Corgi	11 y	♀		-	-	-	C/C	-	-	-	-	-	-
Golden retriever	5 y	♀		-	-	-	C/C	-	-	-	-	-	-
Golden retriever	7 y	♀		-	-	-	A/C	-	-	-	-	-	-
Golden retriever	11 y	♀		-	-	-	C/C	-	-	-	-	-	-

Golden retriever	3 y	♀		-	-	-	C/C	-	-	-	-	-	-
Golden retriever	8 y	♂		-	-	-	C/C	-	-	-	-	-	-
Golden retriever	0 y	♂		-	-	-	A/C	-	-	-	G/G	-	-
Golden retriever	4 y	♀	✓	-	-	-		-		-	-	-	-
Irish Water Spaniel	7 y	♂		-	-	-	A/C	-	-	-	-	-	-
Labrador Retriever	5 y	♂		-	-	-	A/C	-	-	-	-	-	-
Labrador Retriever	10 y	♂		-	-	-	A/C	-	-	-	-	-	-
Labrador Retriever	12 y	♀		-	-	-	-	-	-	-	G/G	-	-
Maltese	10 y	♂		-	-	-	-	-	-	-	A/G	-	-
Miniature Dachshund	5 y	♂	✓	-	-	-	A/C	-	-	-	-	-	-
Miniature Dachshund	10 y	♂		-	-	-	C/C	-	-	-	-	-	-
Miniature Dachshund	0 y	♂		-	-	-	A/C	-	-	-	-	-	-
Miniature Dachshund	5 y	♂		-	-	-	A/C	-	-	-	-	G/A	-
Miniature Dachshund	10 y	♂		-	-	-	A/C	-	-	-	-	-	-
Miniature Dachshund	8 y	♂		-	-	-	C/C	-	-	-	-	-	-
Miniature Dachshund	8 y	♂		-	-	-	-	-	-	-	-	-	-
Miniature Dachshund	9 y	♀	✓	-	-	-	A/C	-	-	-	-	-	-
Miniature Dachshund	7 y	♂					-	-	-	-	-	-	-
Miniature Pinscher	12 y	♀	✓	-	-	-	-	-	-	-	-	-	-
Miniature Schnauzer	14 y	♂		-	A/G	-	-	A/G	-	-	-	-	A/G
Miniature Schnauzer	9 y	♀		-	G/G	-	-	G/G	-	-	-	-	G/G

Papillon	1 y	♂		-	-	-	-	-	-	-	G/G	-	-
Papillon	7 y	♀		-	-	-	C/C	-	-	-	-	-	-
Papillon	7 y	♀	✓	-	-	-	-	-	-	-	-	-	-
Papillon	10 y	♂		-	-	-	A/C	-	-	-	A/G	-	-
Pomeranian	12 y	♀		-	-	-	-	-	-	-	G/G	-	-
Pug	Unknown	♀		-	-	-	-	-	-	-	-	-	-
Pug	4 y	♀		-	-	-	C/C	-	-	-	-	-	-
Pulley	12 y	♀		-	-	-	-	-	-	-	A/G	-	-
Pulley	6 y	♀		-	-	-	-	-	-	-	A/G	-	-
Shetland Sheepdog	10 y	♀		-	-	-	A/C	-		-	-	-	-
Shiba	5 y	♂		-	-	-	C/C	-	-	-	-	-	-
Shiba	10 y	♀		-	-	-	-	-	-	-	-	-	-
Shiba	11 y	♂		-	-	-	C/C	-	-	-	-	-	-
Shiba	4 y	♀		-	-	-	C/C	-	-	-	-	-	-
Shiba	4 y	♀		-	-	-	C/C	-	-	-	-	-	-
Shih Tzu	10 y	♀	✓	-	-	-	-	-	-	A/C	-	-	-
Shih Tzu	7 y	♀	✓	G/A	-	-	-	-	T/G	-	-	-	-
Shih Tzu	8 y	♂		-	-	-	-	-		-	-	-	-
Siberian Husky	13 y	♀		-	-	-	-	-	-	-	G/G	-	-
West Highland White Terrier	4 y	♀		-	-	-	A/C	-	-	-	-	-	-
Mix	13 y	♀		-	-	-	-	-	-	-	-	-	-

Mix	9 y	♀	✓	-	-	-	-	-	-	A/C	-	-	-
Mix	7 y	♀	✓	-	A/G	-	-	A/G	T/G	-	-	-	A/G
Mix	14 y	♂		-	-	-	-	-	-	-	G/G	-	-
Mix	9 y	♀	✓	-	-	-	-	-	-	-	G/G	-	-
Mix	10 y	♂	✓	-	-	-	A/C	-	-	A/C	-	-	-
Mix	7 y	♀	✓	-	-	-	A/C	-	-	-	A/G	-	-
Mix	12 y	♂		-	-	-	C/C	-	-	-	-	-	-
Mix	10 y	♂		-	-	-	C/C	-	-	-	-	-	-
Mix	15 y	♂		G/A	-	C/T	A/C	-	-	-	-	-	-
Mix	12 y	♂		-	-	-	-	-	-	-	-	-	-
Mix	11 y	♂		-	-	-	C/C	-	-	-	-	-	-
Mix	13 y	♂					C/C	-	-	-	-	-	-
Mix	14 y	♀	✓	-	-	-	-	-	-	-	-	-	-
Mix	10 y	♂	✓	-	-	-	C/C	-	-	-	-	-	-
Mix	11 y	♀	✓	-	-	-	A/C	-	-	-	-	-	-
Mix	10 y	♀		-	-	-	C/C	-	-	-	-	-	-
Mix	9 y	♂	✓	-	-	-	A/C	-	-	-	A/G	-	-
Mix	10 y	♂		-	-	-	-	-	-	-	-	-	-
Mix	4 y	♀		-	-	-	A/C	-	-	-	-	-	-
Mix	4 y	♀		-	-	-	-	-	-	-	-	-	-
Mix	4 y	♀		-	-	-	-	-	-	-	-	-	-
