# Supplementary material for: A Highly Conserved Region in BRCA2 Suppresses the RAD51-Interaction Activity of BRC Repeats
Source: Vet Sci. 2023 Feb 10;10(2):145. doi: 10.3390/vetsci10020145 (PMC9959916; doi:10.3390/vetsci10020145)
Supplement: Supplementary file 1 [file vetsci-10-00145-s001.zip › Supplementary Figure S1.pdf]

(A)

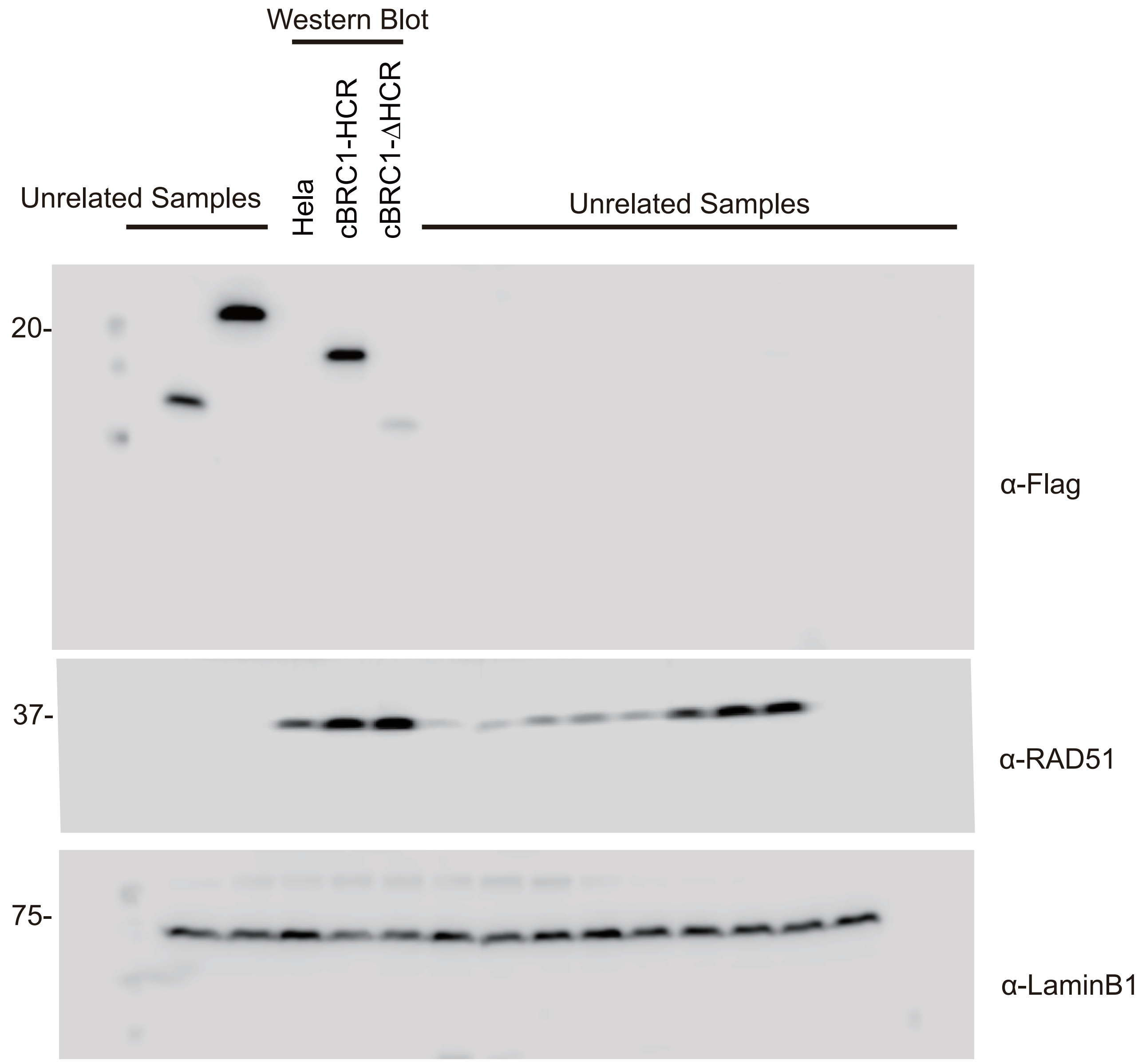

Western Blot
Unrelated Samples
Hela
cBRC1-HCR
cBRC1-ΔHCR
Unrelated Samples
20-
37-
75-
α-Flag
α-RAD51
α-LaminB1

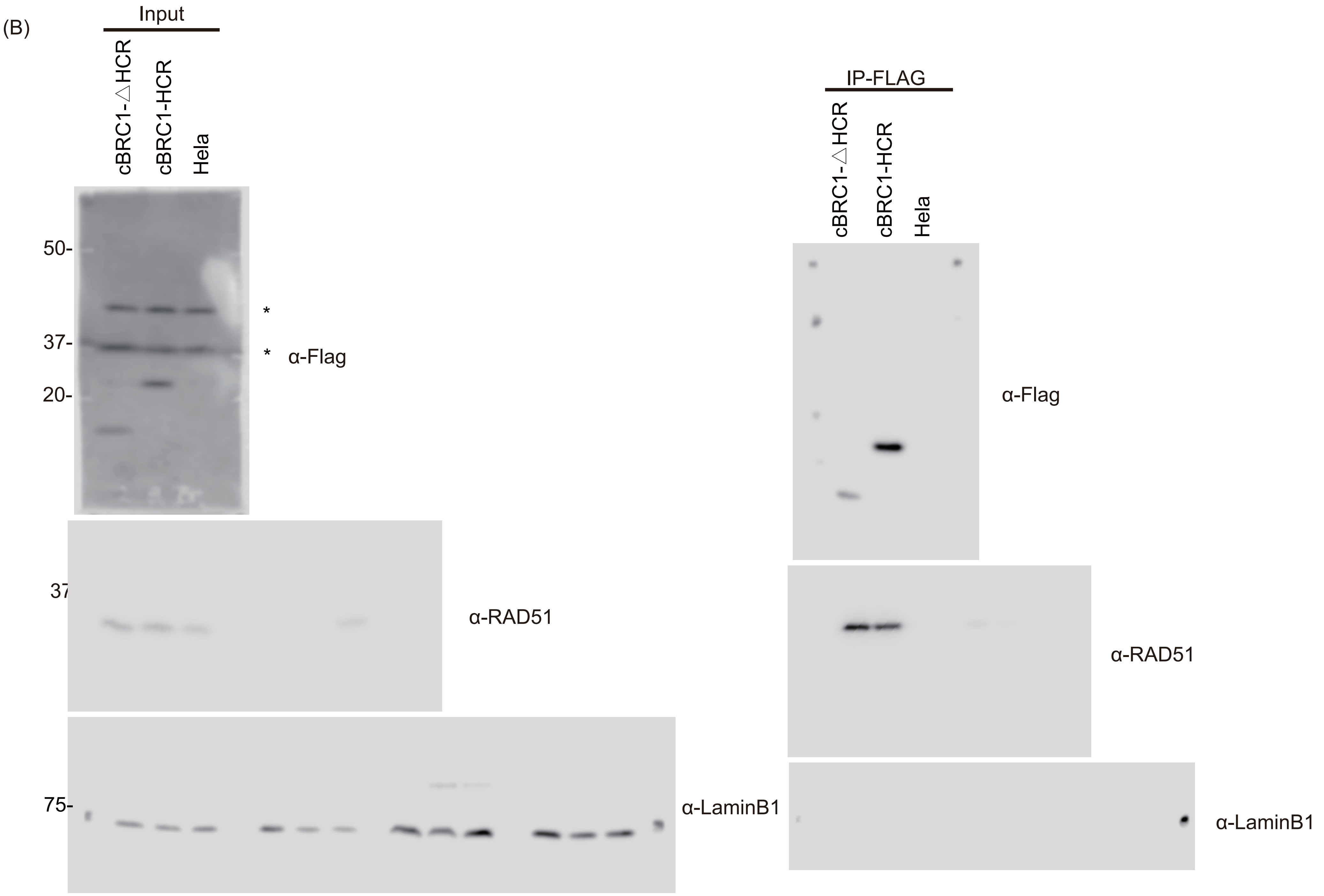

Asterisks (*) indicate indicate nonspecific bands

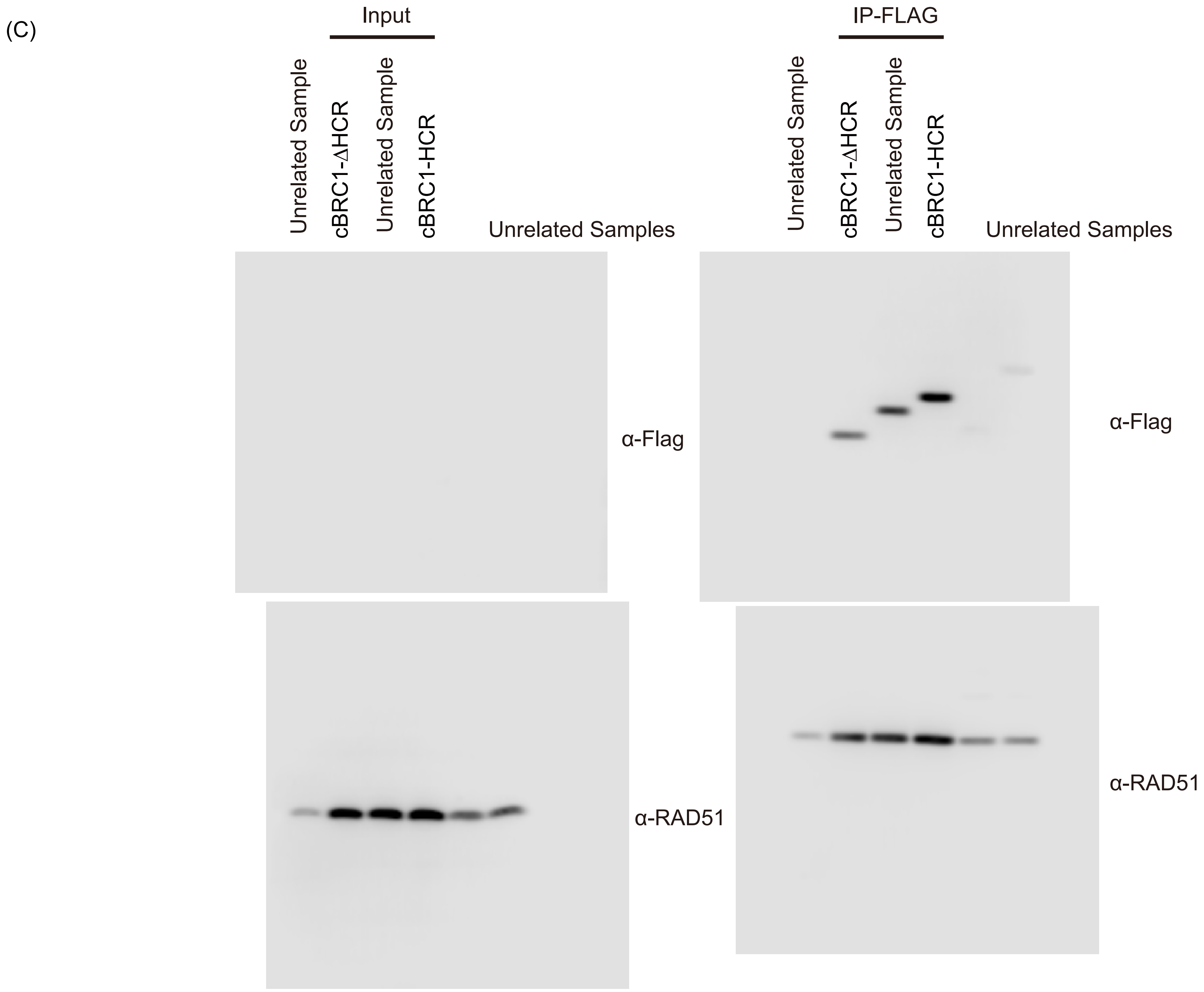

(C)
Input
IP-FLAG
Unrelated Sample
cBRC1-ΔHCR
Unrelated Sample
cBRC1-HCR
Unrelated Samples
Unrelated Sample
cBRC1-ΔHCR
Unrelated Sample
cBRC1-HCR
Unrelated Samples
α-Flag
α-Flag
α-RAD51
α-RAD51

(D)

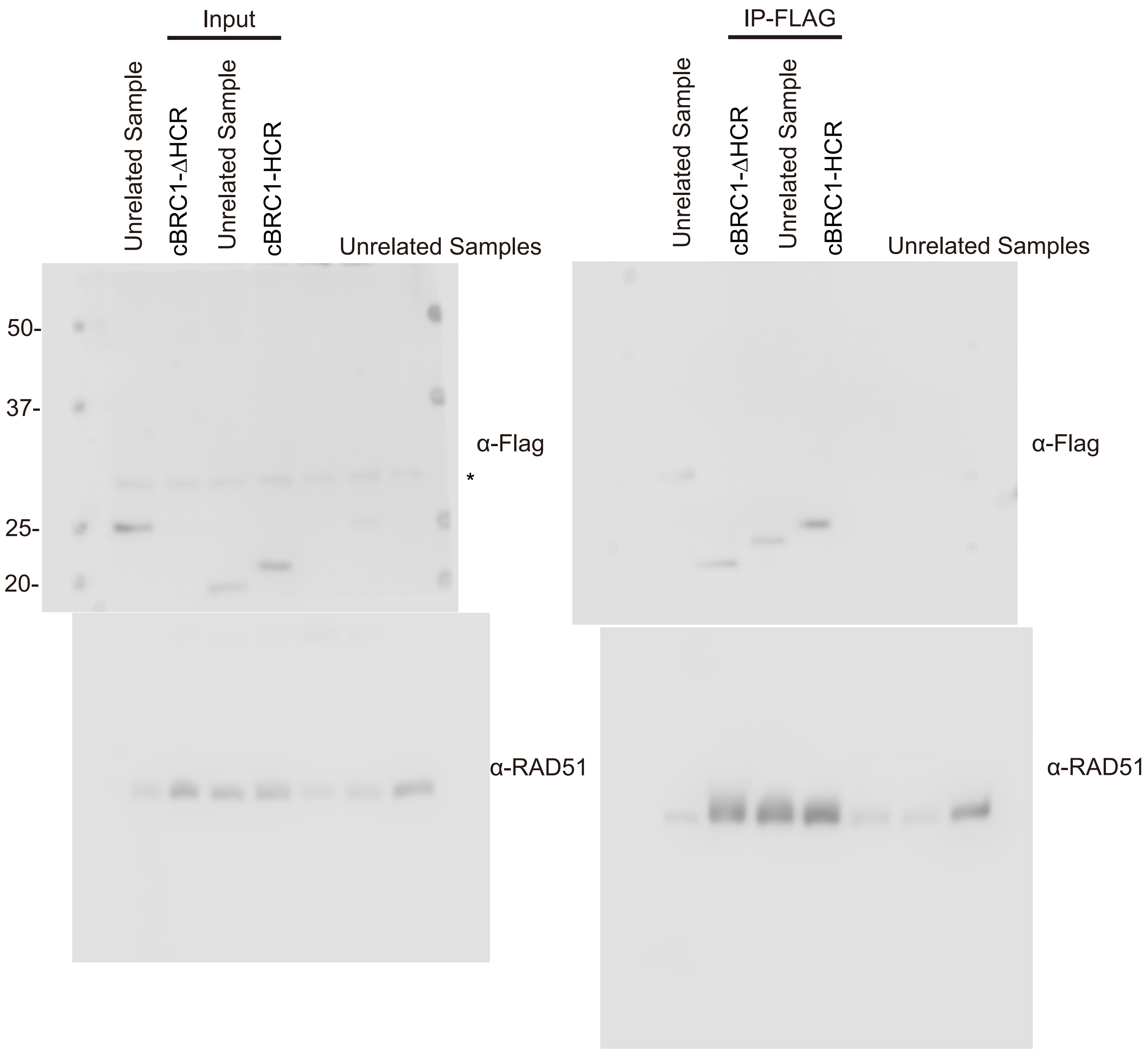

Asterisks (*) indicate nonspecific bands.

(E)

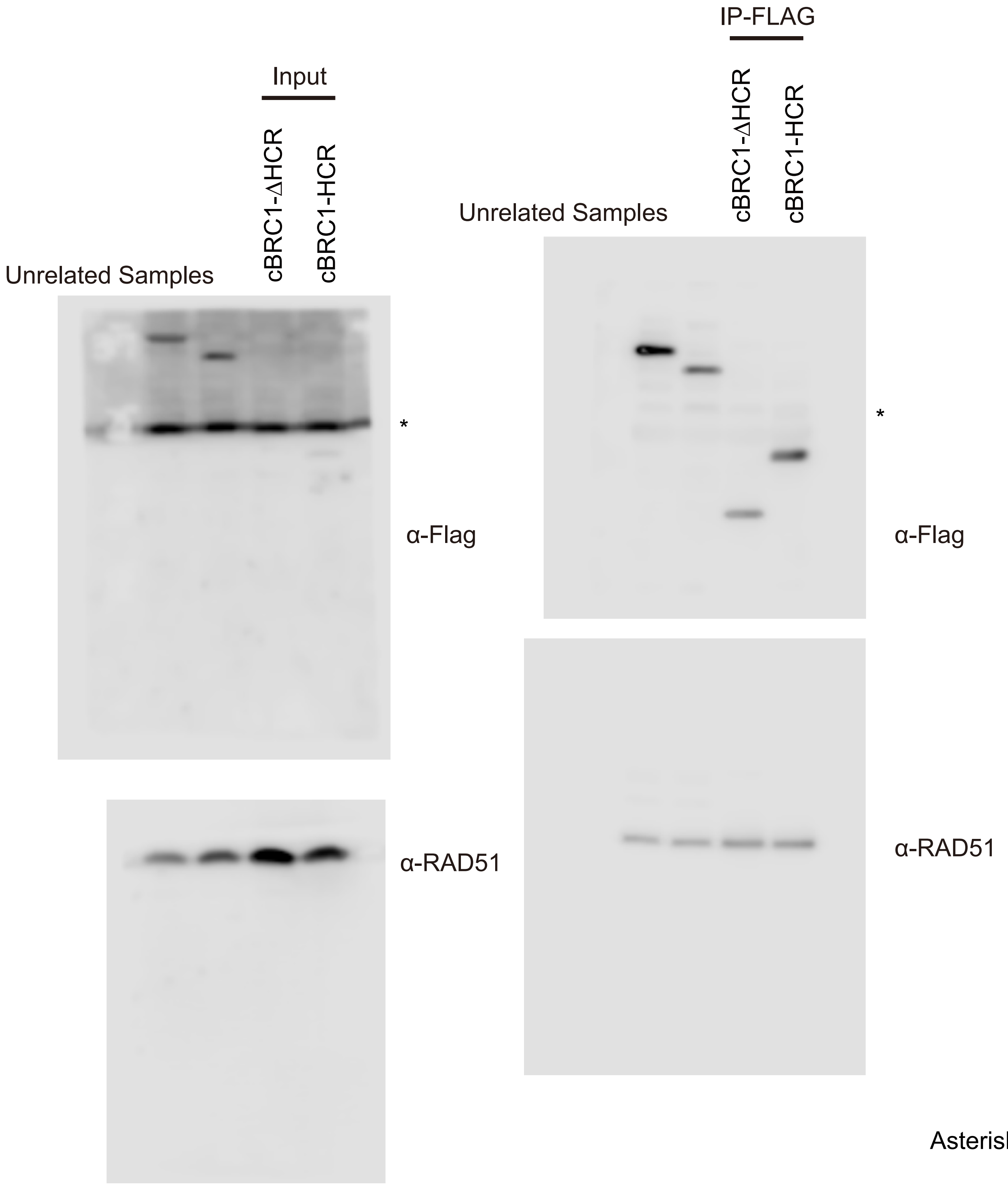

Asterisks (*) indicate nonspecific bands.

(F)

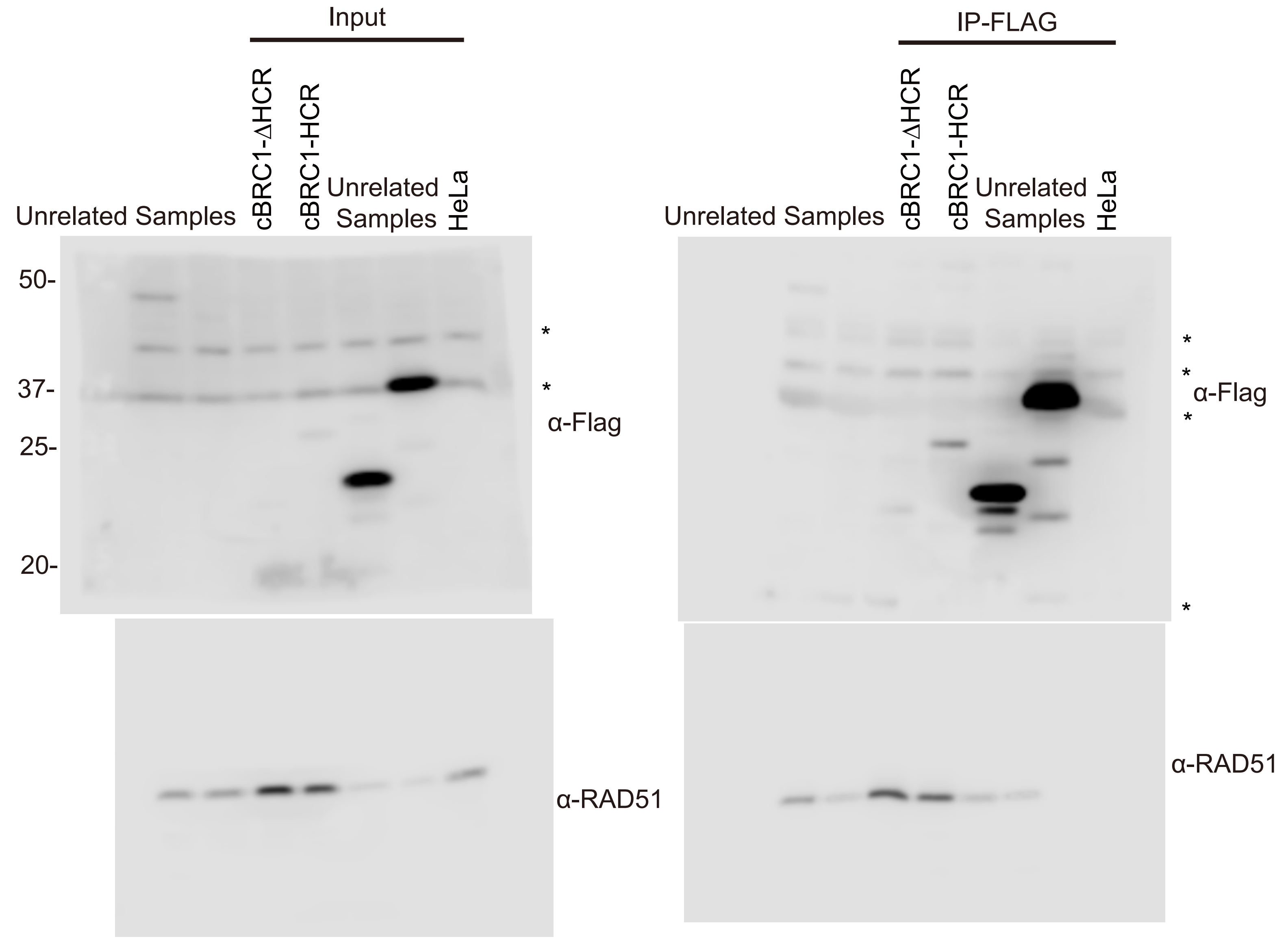

Asterisks (*) indicate nonspecific bands.

(G)

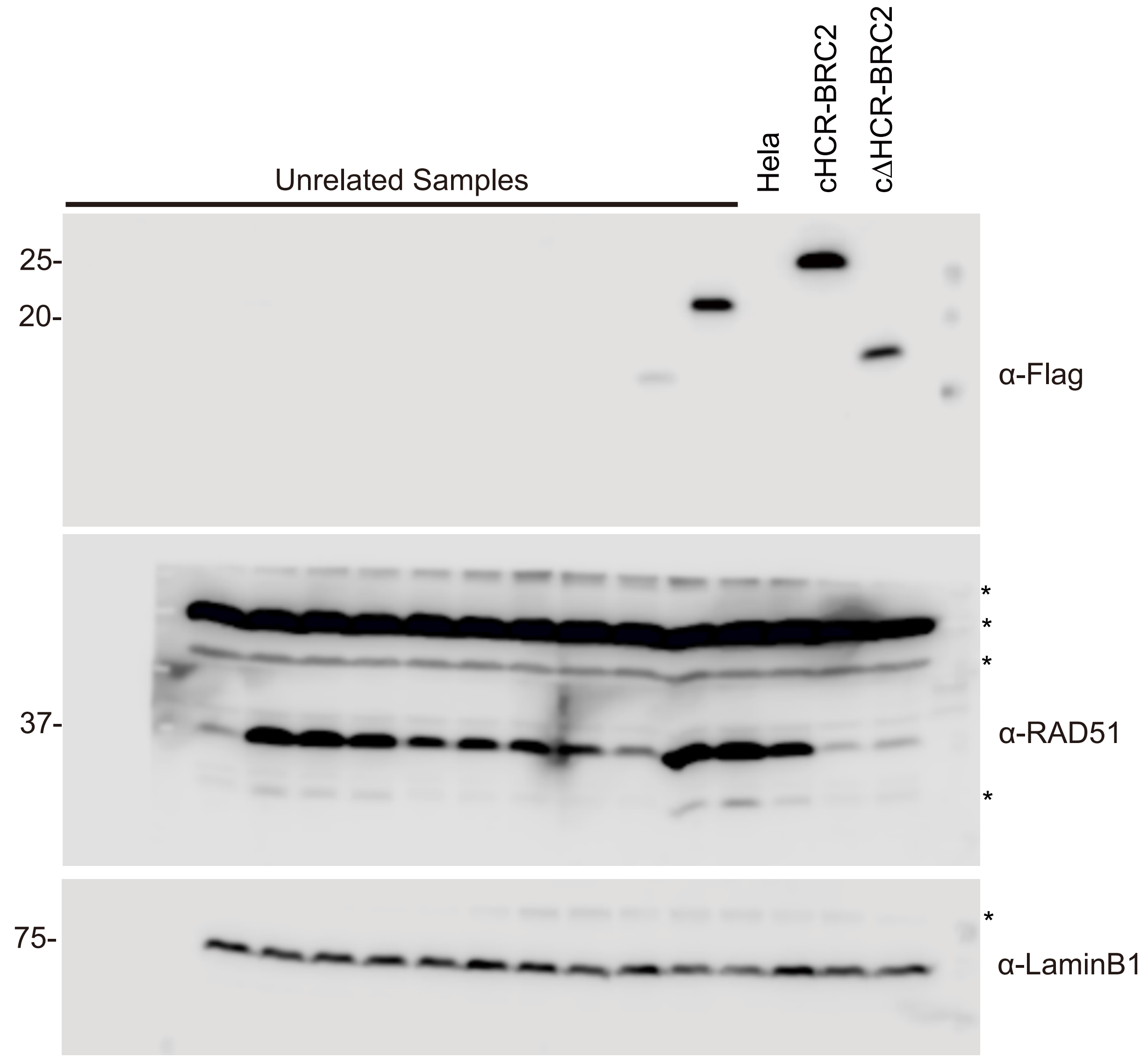

Asterisks (*) indicate indicate unrelated bands or nonspecific bands

(H)

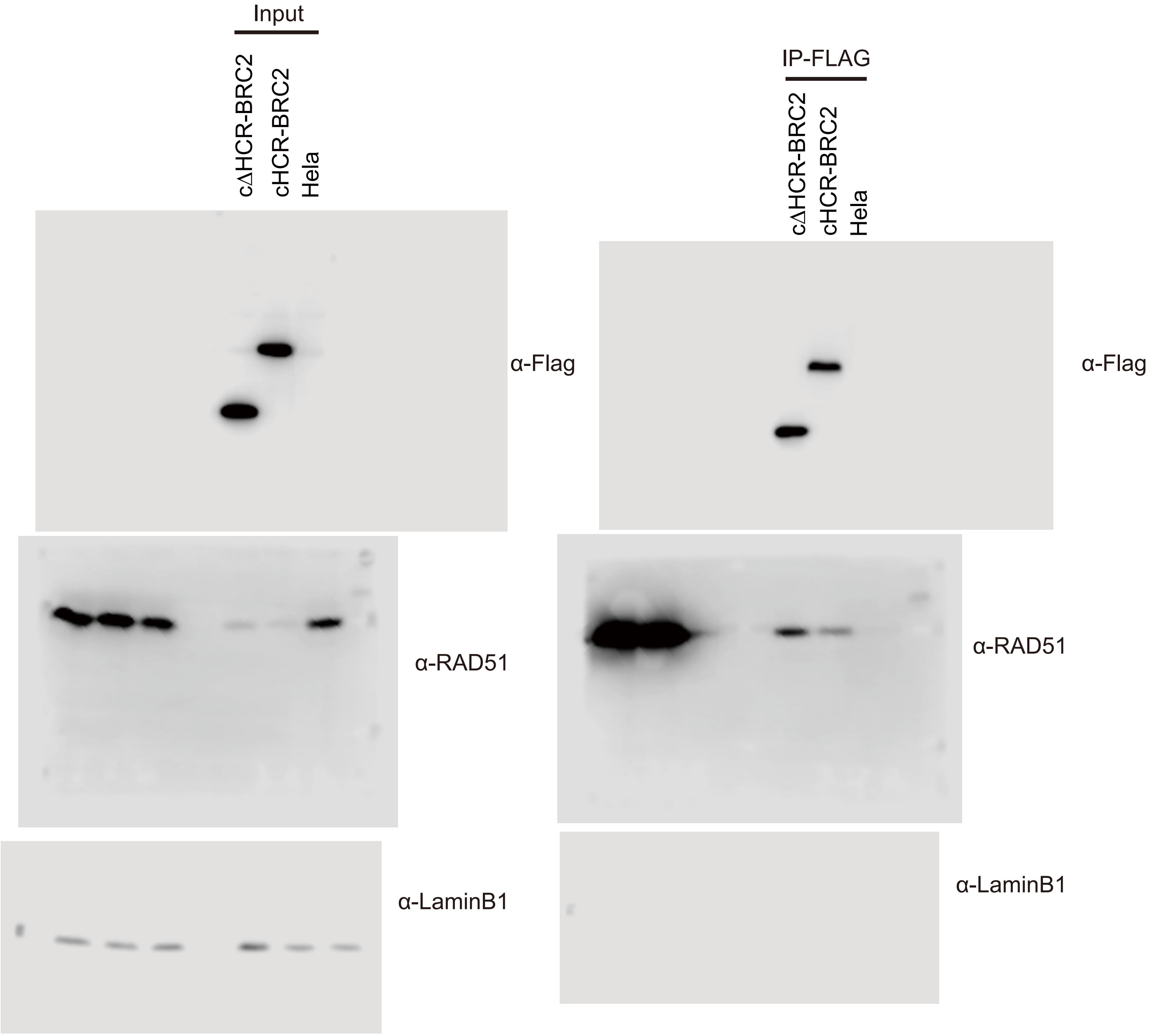
Input
cΔHCR-BRC2
cHCR-BRC2
Hela
IP-FLAG
cΔHCR-BRC2
cHCR-BRC2
Hela
α-Flag
α-Flag
α-RAD51
α-RAD51
α-LaminB1
α-LaminB1

(I)

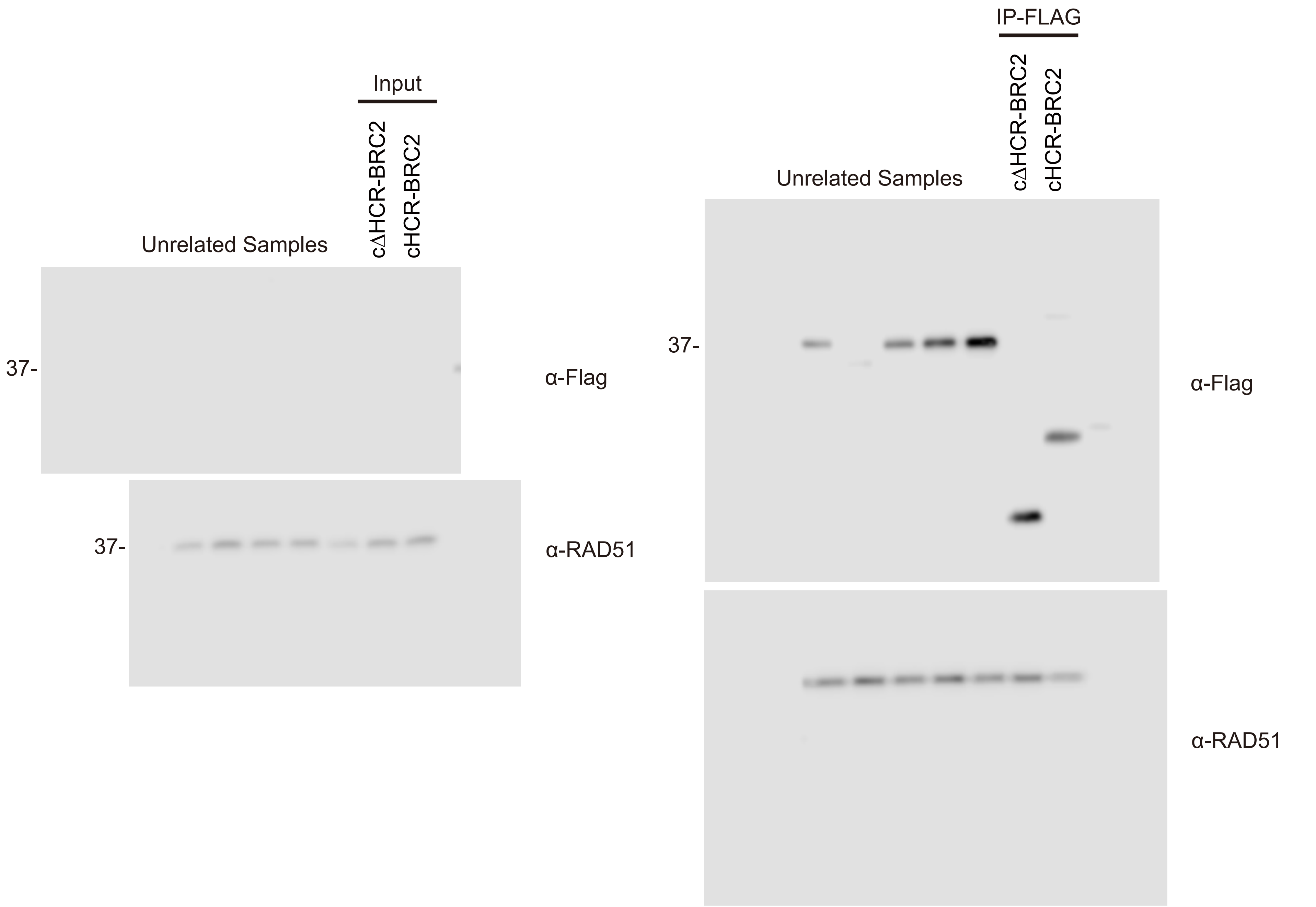

Input
cΔHCR-BRC2
cHCR-BRC2
Unrelated Samples
37-
α-Flag
37-
α-RAD51
IP-FLAG
cΔHCR-BRC2
cHCR-BRC2
Unrelated Samples
37-
α-Flag
α-RAD51

(J)

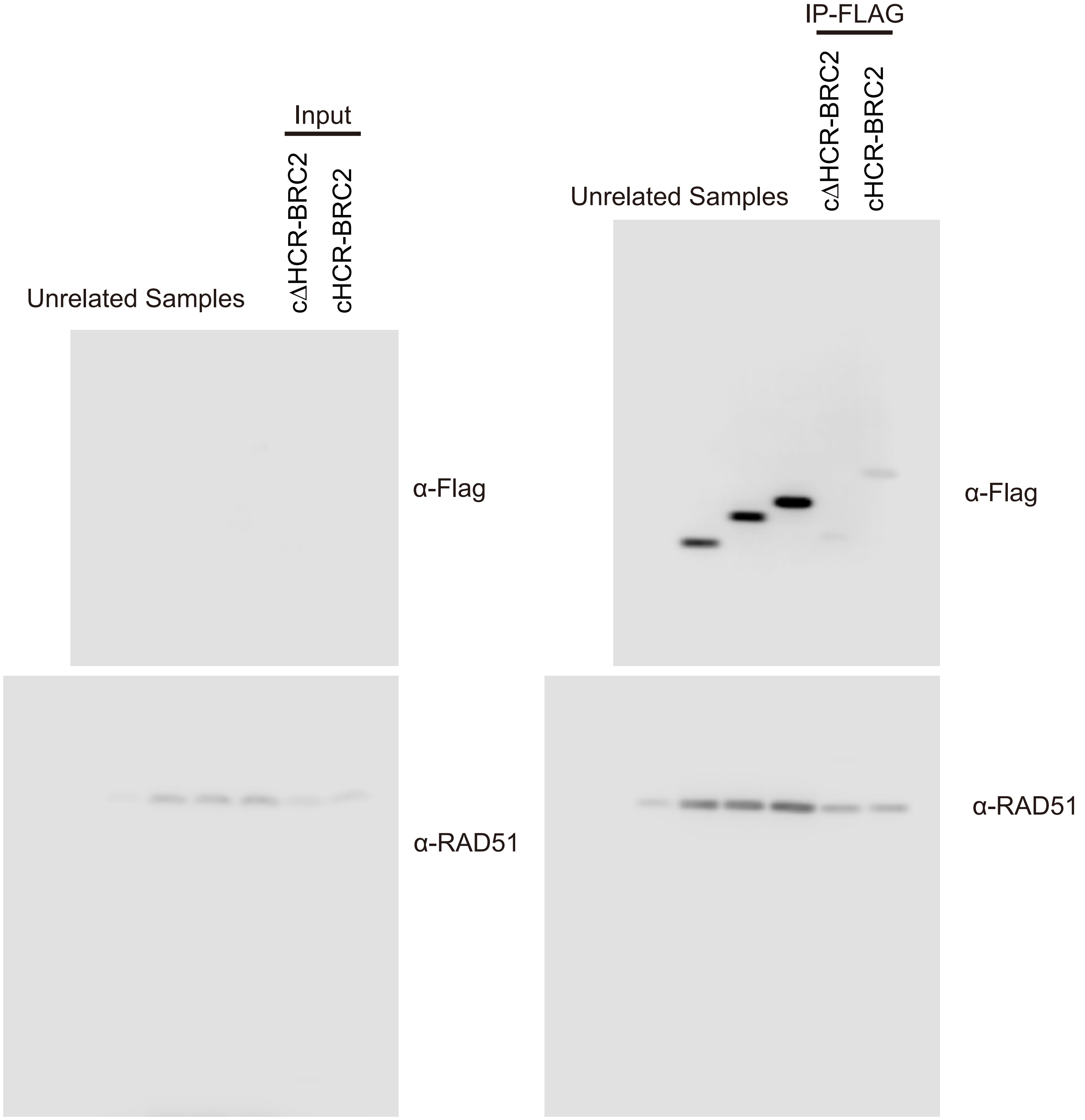

Asterisks (*) indicate nonspecific bands.

(K)

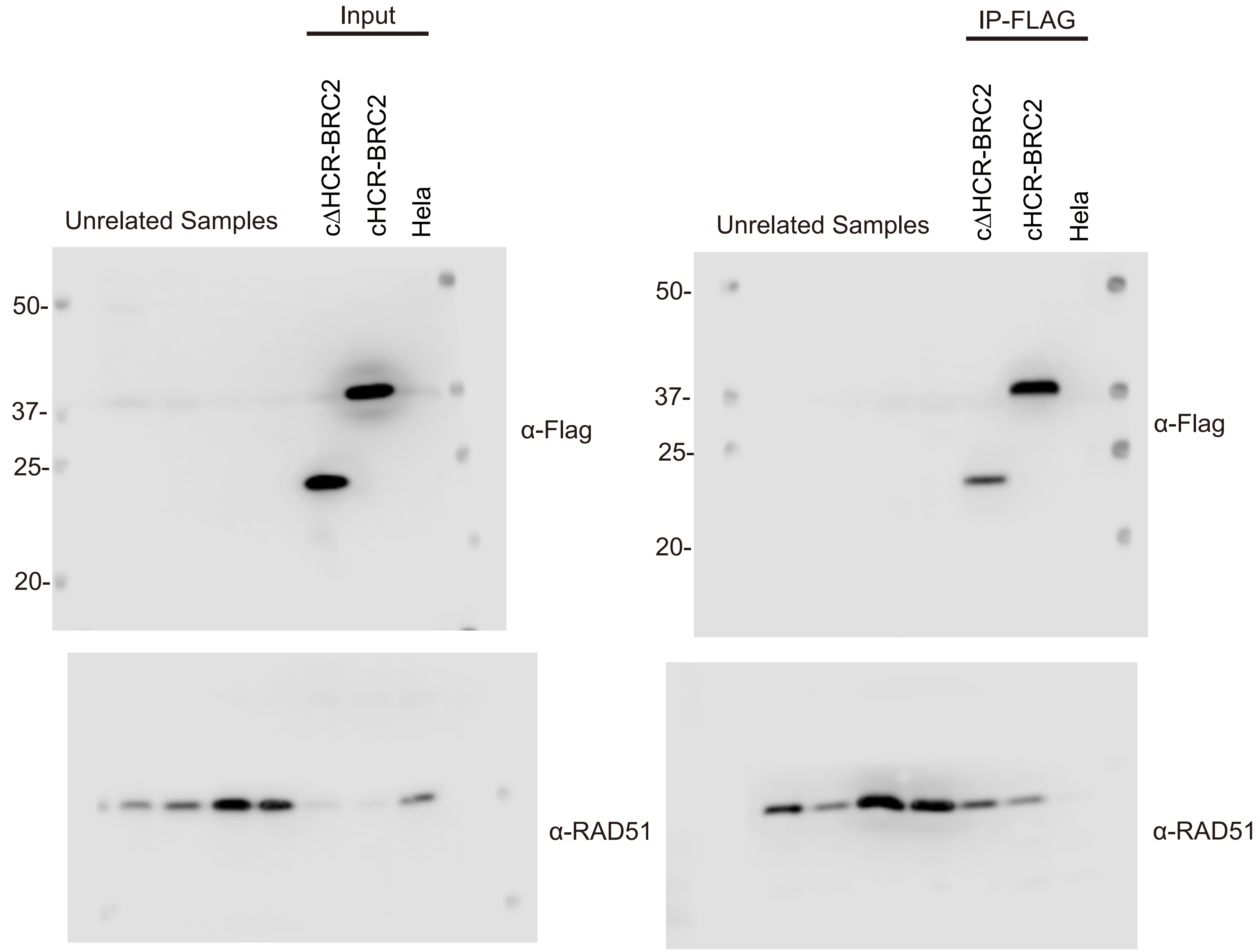
Input
IP-FLAG
Unrelated Samples
cΔHCR-BRC2
cHCR-BRC2
Hela
Unrelated Samples
cΔHCR-BRC2
cHCR-BRC2
Hela
50-
37-
25-
20-
α-Flag
50-
37-
25-
20-
α-Flag
α-RAD51
α-RAD51

(L)

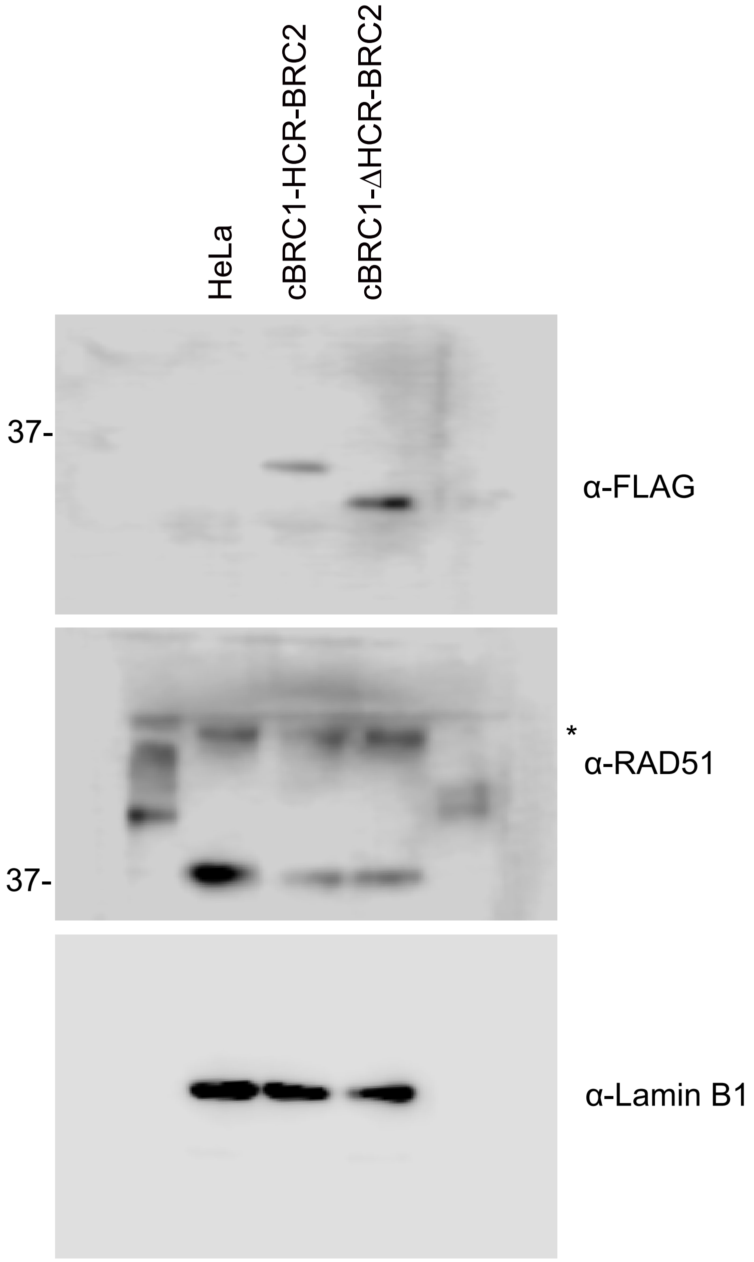

Asterisks (*) indicate indicate unrelated bands or nonspecific bands

(M)

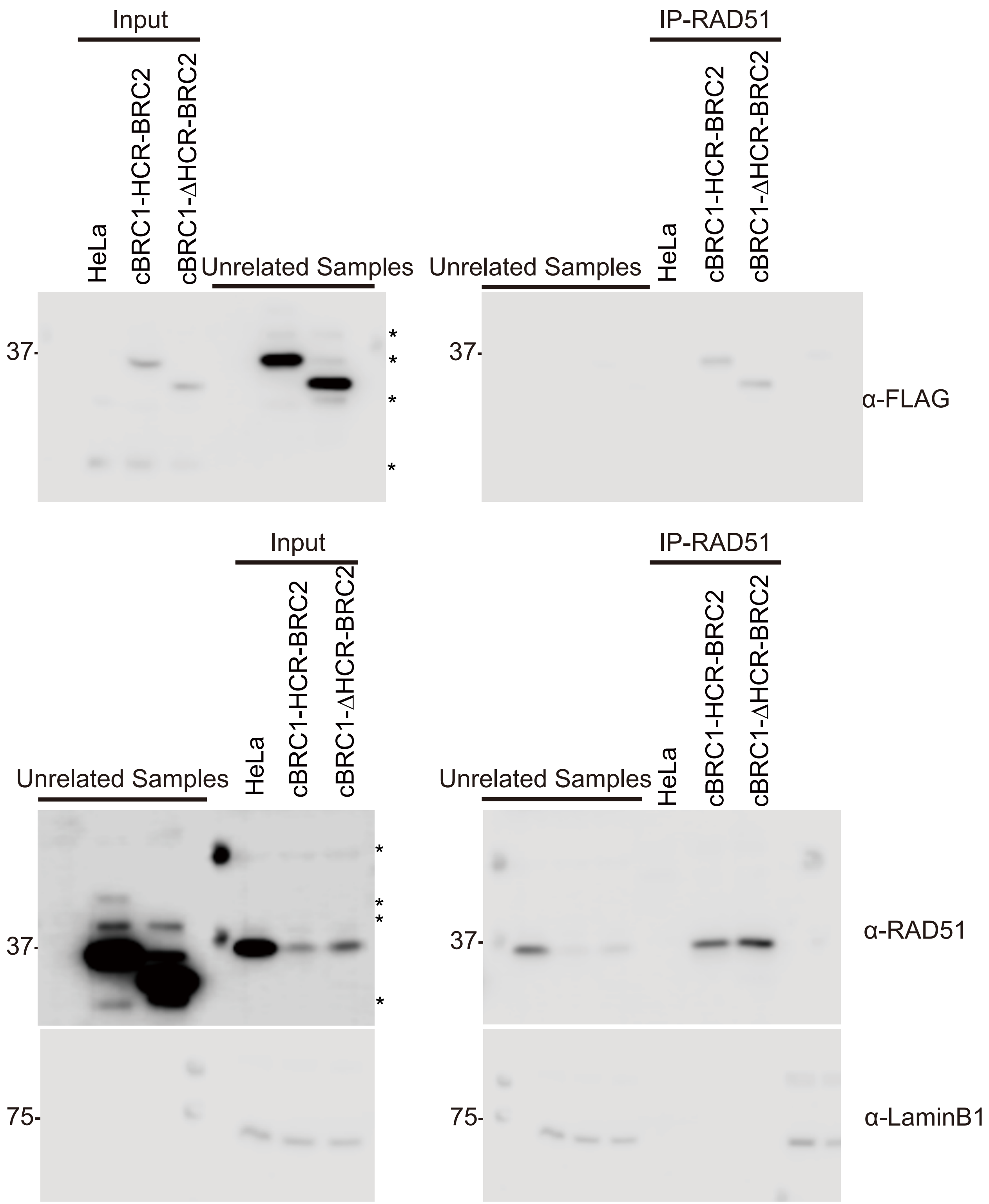

Asterisks (*) indicate indicate unrelated bands or nonspecific bands

(N)

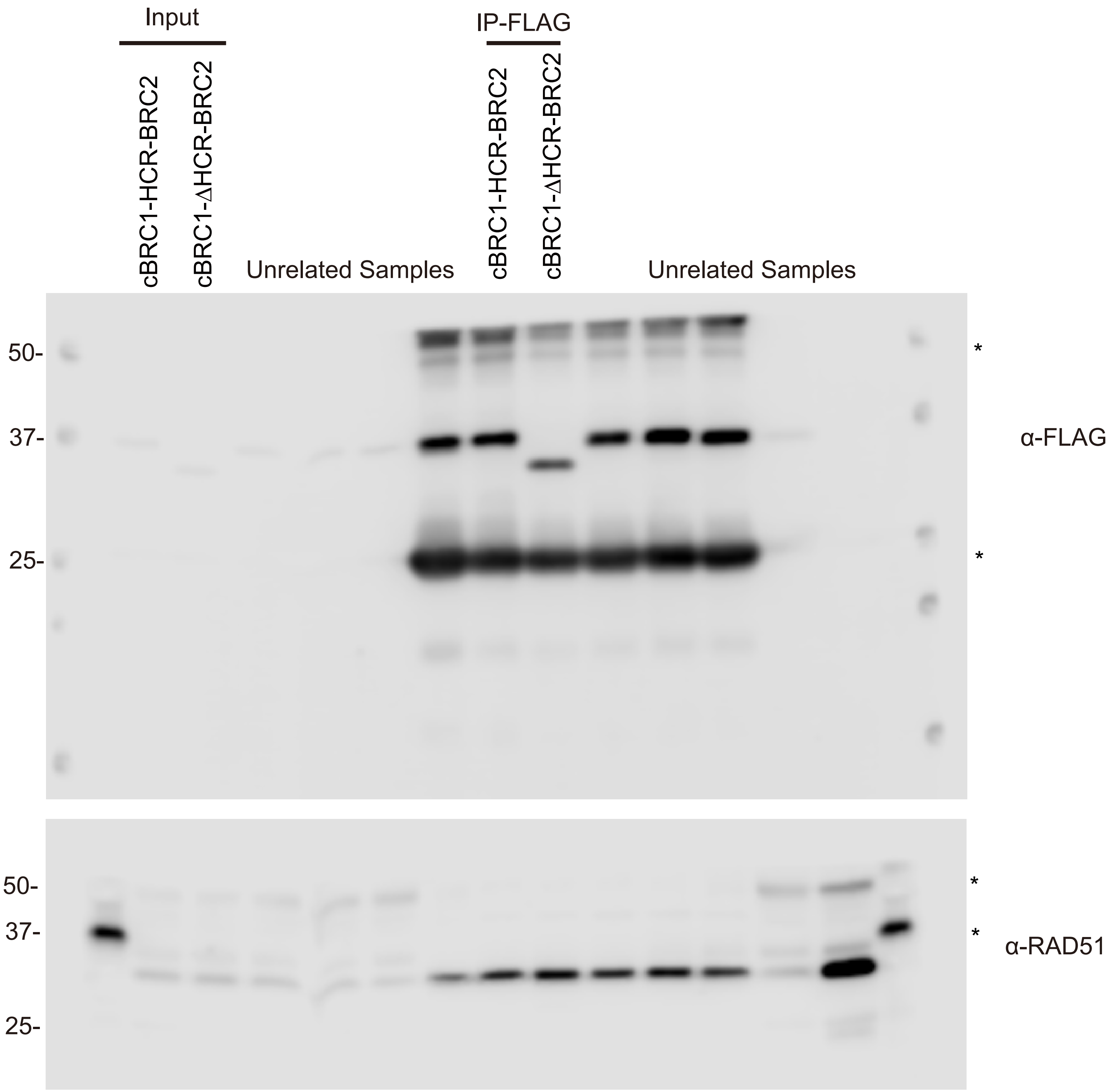

Asterisks (*) indicate indicate IgG heavy chain and light chain bands or nonspecific bands

(O)

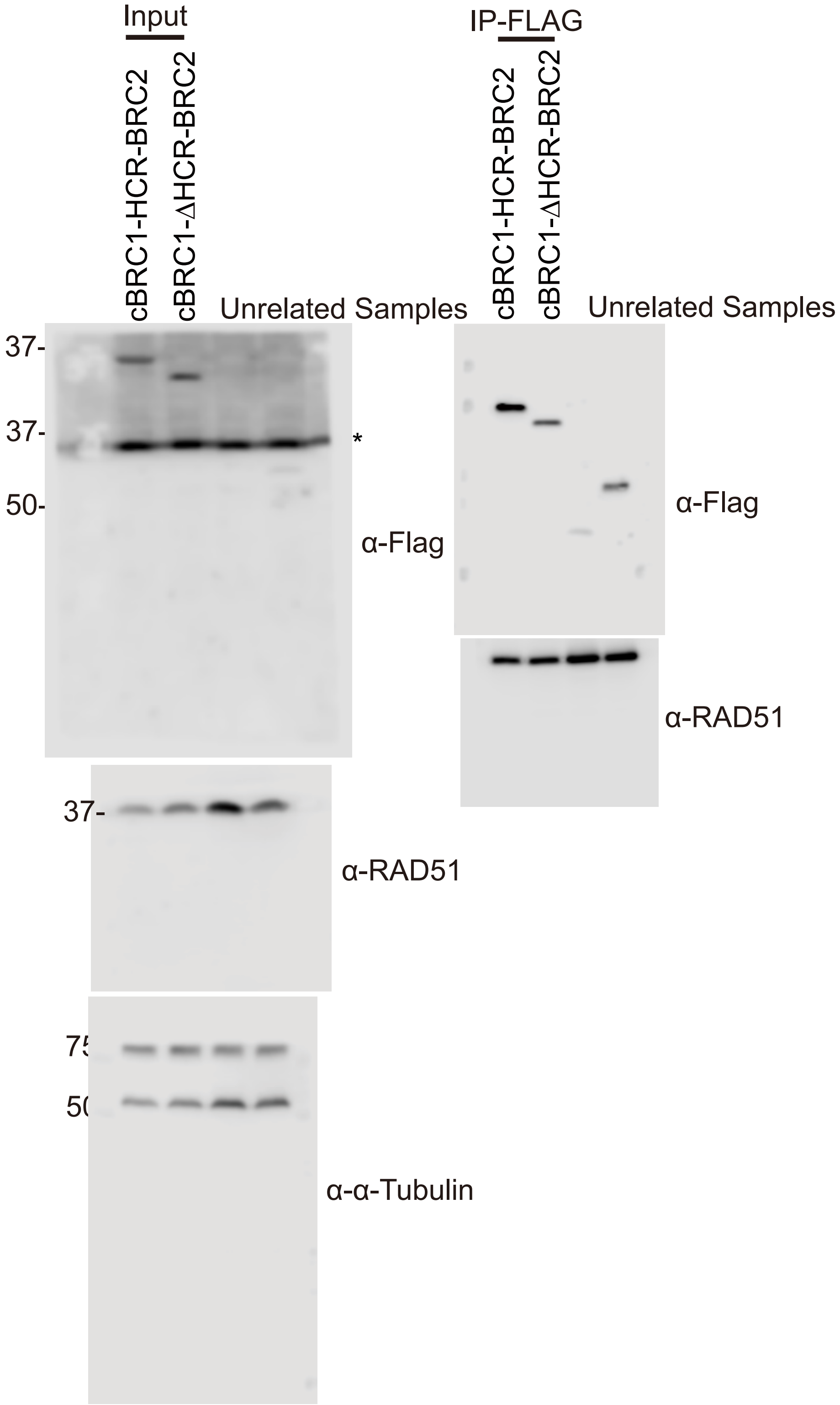

Asterisks (*) indicate nonspecific bands.

(P)

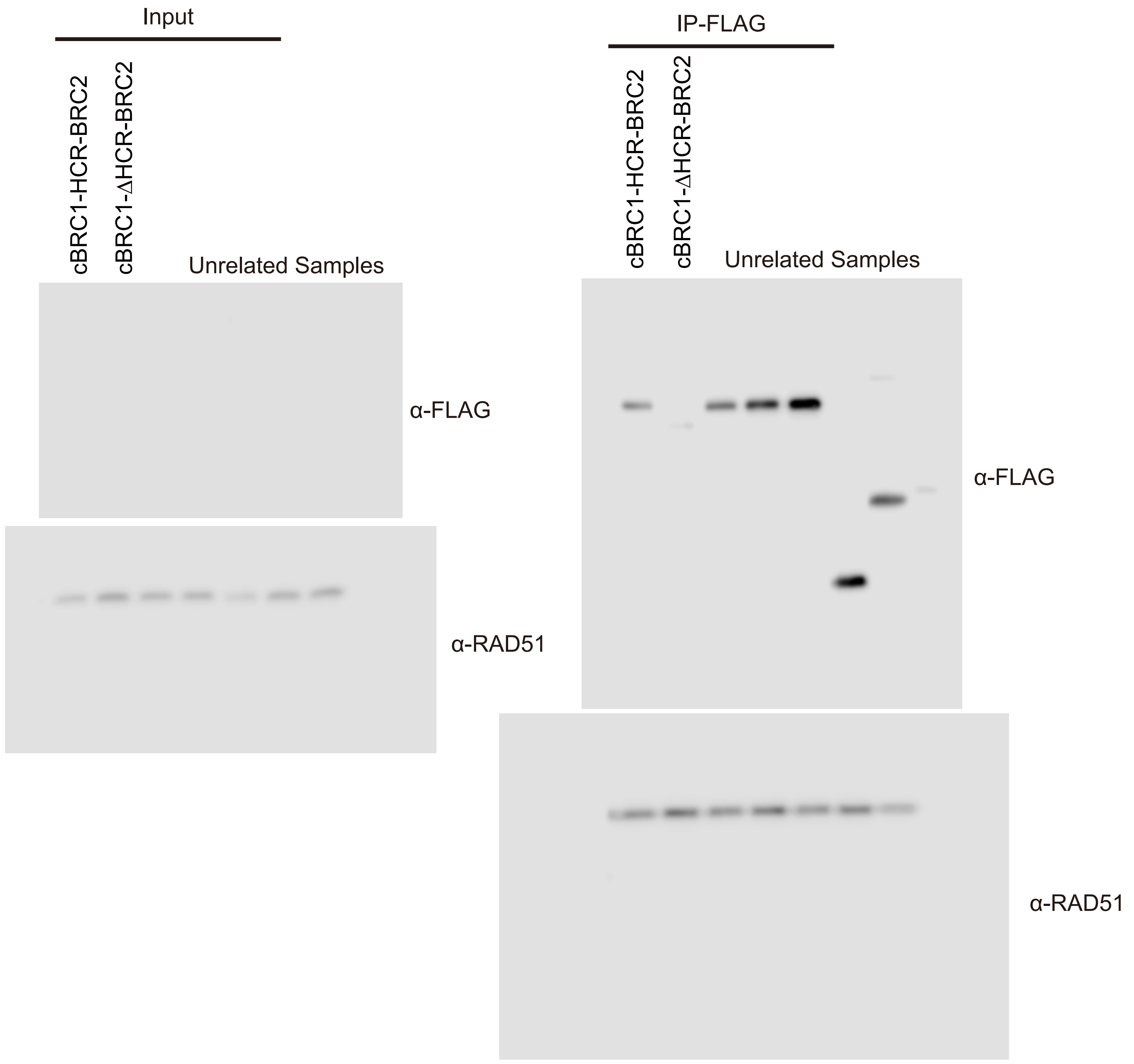
Input
IP-FLAG
cBRC1-HCR-BRC2
cBRC1-ΔHCR-BRC2
Unrelated Samples
α-FLAG
α-RAD51

(Q)

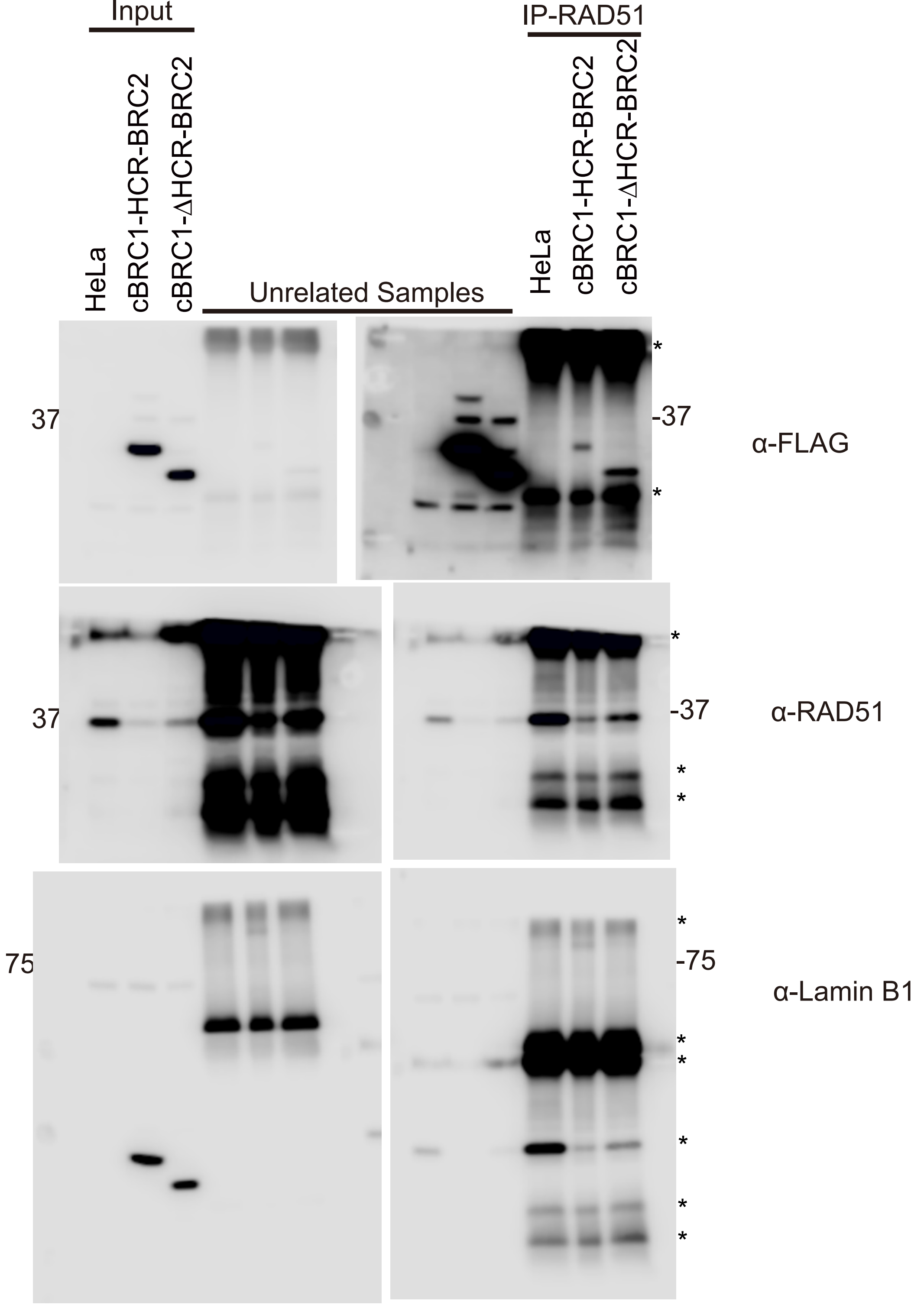

Asterisks (*) indicate indicate IgG heavy chain and light chain bands, unrelated bands, or nonspecific bands

(R)

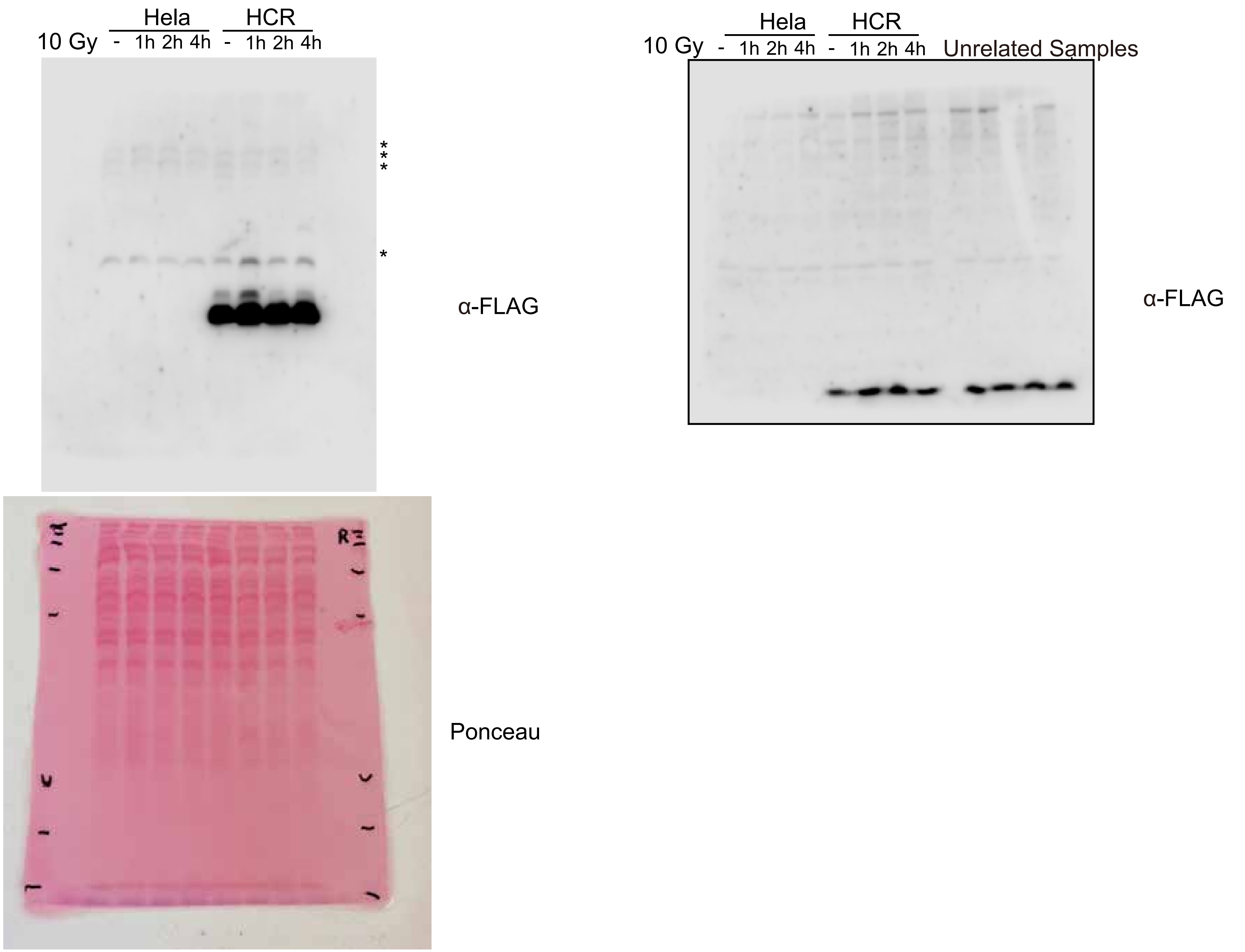

Asterisks (*) indicate nonspecific bands.

(S)

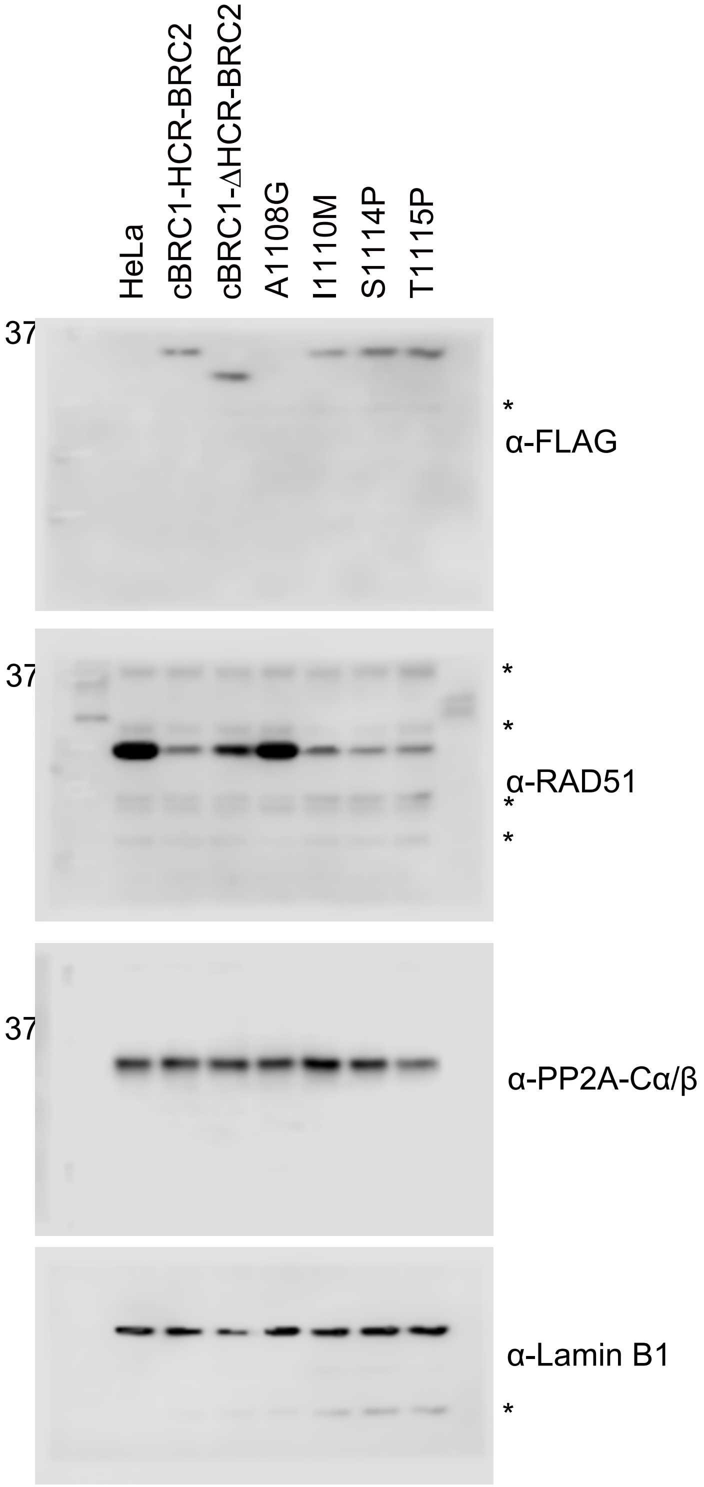

*

Asterisks (*) indicate indicate nonspecific bands

(T)

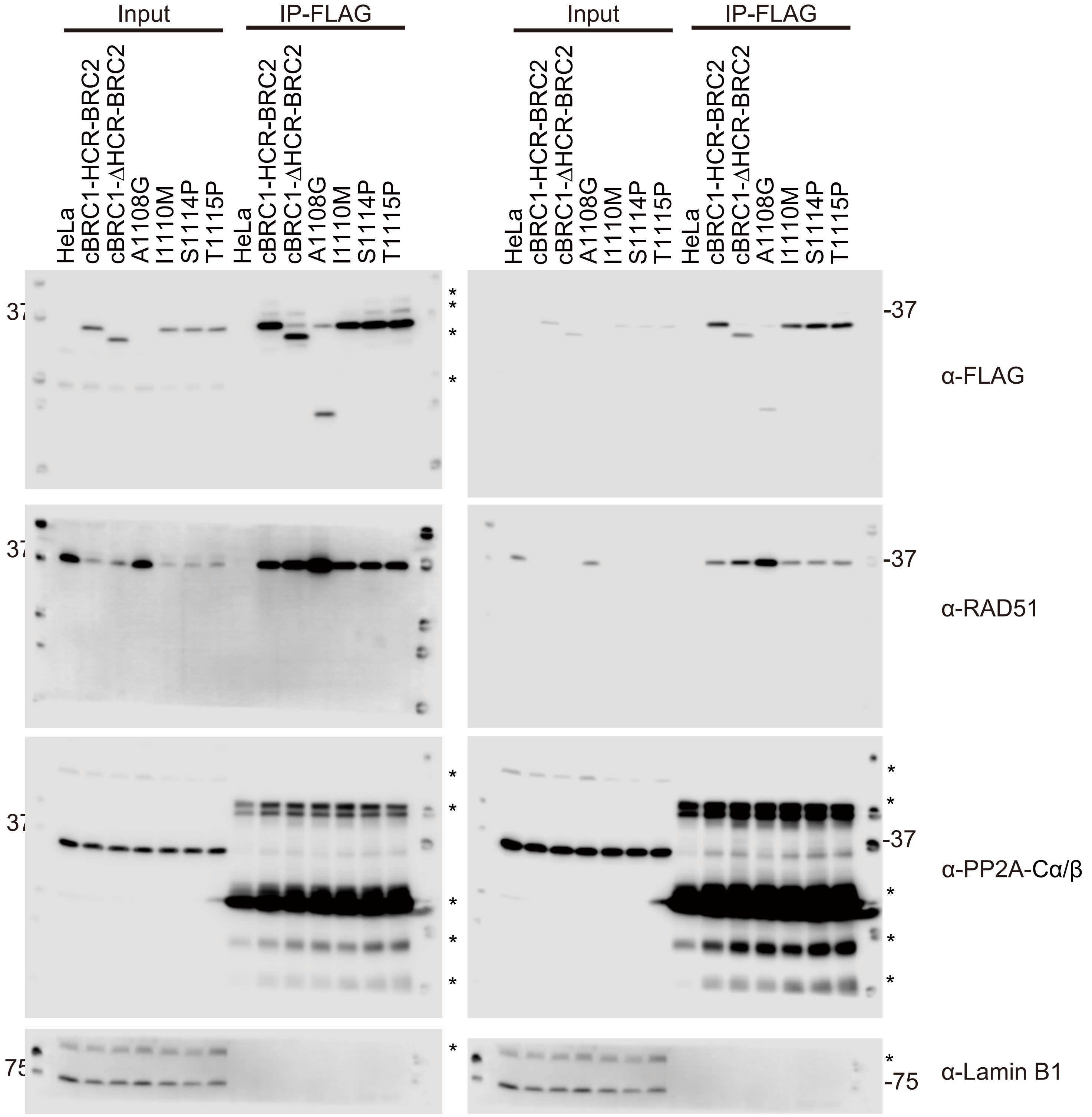

Asterisks (*) indicate indicate IgG heavy chain and light chain bands, unrelated bands, or nonspecific bands

Figure S1. Original Western blot images.

(A) Original Western blot images of Figure 2A.
(B) Original Western blot images of Figure 2B and quantitative analysis of Figure 2C.
(C-F) Original Western blot images of quantitative analysis of Figure 2C.
(G) Original Western blot images of Figure 2D.
(H) Original Western blot images of Figure 2E and quantitative analysis of Figure 2F.
(I-K) Original Western blot images of quantitative analysis of Figure 2F.
(L) Original Western blot images of Figure 3A.
(M) Original Western blot images of Figure 3B and quantitative analysis of Figure 3C.
(N-P) Original Western blot images of quantitative analysis of Figure 3C.
(Q) Original Western blot images of Figure 3D.
(R) Original Western blot images of Figure 4B.
(S) Original Western blot images of Figure 5B.
(T) Original Western blot images of Figure 5C.
